# Overall survival in multiple myeloma in Brazil: A cohort of 16 years

**DOI:** 10.1016/j.htct.2025.103962

**Published:** 2025-09-12

**Authors:** Deborah Marta do Santos Oliveira, Isabela Cristina Menezes de Freitas, Wallace Mateus Prata, Isabella Zuppo Laper, Pâmela Santos Azevedo, Adriano de Paula Sabino, Marisa Yurico Itonaga, Carmino Antonio de Souza, Mariângela Leal Cherchiglia, Juliana Alvares Teodoro, Francisco de Assis Acurcio, Augusto Afonso Guerra Junior

**Affiliations:** a Faculty of Pharmacy, Federal University of Minas Gerais, Belo Horizonte, Minas Gerais, Brazil; b Collaborating Centre for Technology Assessment in Health and Excellence (CCATES), Federal University of Minas Gerais, Belo Horizonte, Minas Gerais, Brazil; c Research and Development Directorate of the Ezequiel Dias Foundation, Minas Gerais, Belo Horizonte, Minas Gerais, Brazil; d Health Consultant Specialized in Oncology, São Paulo, Brazil; e Hematology and Hemotherapy Center, University of Campinas, São Paulo, Brazil; f Department of Preventive and Social Medicine at the Federal University of Minas Gerais, Belo Horizonte, Minas Gerais, Brazil

**Keywords:** Multiple myeloma, Survival analysis, Cohort studies, Medical record linkage, Real world evidence

## Abstract

Multiple myeloma constitutes approximately 1 % of all malignancies, with a higher incidence observed in over 65-year-old individuals. New technologies have shown promising results with an increased overall survival. The objective of this cohort study was to evaluate the survival analysis of patients with multiple myeloma treated by the Brazilian Unified Health Service over 16 years and compare the effectiveness of bortezomib (Bortezomib)-based treatment with other regimens used. A retrospective national cohort study was conducted utilizing real-world evidence derived from the Brazilian Unified Health System big data. This study focused on 25,370 patients with multiple myeloma who underwent chemotherapy between 2000 and 2015. Of these patients, 50.71 % were male, and the median age was 62 years. The median overall survival was 37 months. Hematopoietic stem cell transplantation (HSCT) was the best prognostic factor with overall survival of 87 months. The bortezomib (Bortezomib)-based chemotherapy provided the best results of the different chemotherapy regimens in terms of overall survival (67 months), followed by thalidomide-based schemes with an overall survival of 54 months. Despite the significant progress made in the Brazilian health system, the National Committee for Technology Incorporation (CONITEC) needs to make quicker decisions to improve access to new oncology drugs for patients, while maintaining rigorous evaluation criteria. Earlier adoption and adequate funding for oncology services could have saved more lives compared to the treatments made available by the Unified Health Service at that time.

## Introduction

Multiple Myeloma (MM) is a hematological disease characterized by the multiplication of malignant plasma cells in the bone marrow. As the second most common malignant hematological disease after lymphoma, MM represents approximately 10 % of such cases and accounts for 1 % of all types of cancer [1,2].

Demographically, MM predominantly affects elderly individuals, with a mean age at diagnosis of 66 years, and a majority of patients (56 %) being male [3]. The actual incidence of MM in Brazil is unknown according to information available in reports of the National Cancer Institute [4,5]. Data provided by the Institute for Health Metrics and Evaluation show that 1.83 deaths per 100,000 inhabitants occurred in Brazil in 2019 due to MM, whereas data from the United States report 5.47 deaths per 100,000 inhabitants [6].

The diagnosis of MM is characterized by bone marrow clonal plasma cells ≥10 % or bone or extramedullary plasmacytoma proven by biopsy, in addition to one or more of the following: evidence of target organ damage that may be attributed to an underlying plasma cell proliferative disorder, specifically: [C] Hypercalcemia: serum calcium >11 mg/dL or >1 mg/dL above the upper limit of normal; [R] Renal failure: creatinine clearance <40 mL in one minute or serum creatinine >177 mmol/L (>2 mg/dL); [A] Anemia: hemoglobin value <10 g/dL or 2 g/dL below the lower limit of normal; [B] Bone lesions: one or more osteolytic lesions on skeletal radiography, computed tomography (CT) or Positron emission tomography–computed tomography (PET-CT). And one or more of the following biomarkers of malignancy: percentage of plasma cells in the bone marrow biopsy ≥60 %; Ratio of Serum Free Light Chains ≥100; >1 focal lesion in magnetic resonance studies [5,7].

The treatment of symptomatic MM is with drugs, such as chemotherapeutics, immunomodulatory agents, proteasome inhibitors, monoclonal antibodies, and more recently, bispecific antibodies and advanced cell therapy combined or not with radiotherapy. HSCT is an important therapeutic option and may be performed in eligible patients. The goal of treatment is to reach an objective overall response rate (symptom and biochemical control), since it is an incurable disease. Patients experience multiple recurrences until becoming refractory to the treatment [8], leading to death.

In the Brazilian Unified Health System (SUS), the available drugs (bortezomib, cyclophosphamide, cisplatin, dexamethasone, doxorubicin, liposomal doxorubicin, etoposide, melphalan, vincristine and thalidomide) may be used in different combinations [9, 10, 11].

Limited research has been published regarding MM in Brazil, a nation comprising approximately 210 million inhabitants. Most of the reports cover single institution experiences or small numbers of patients compared to this nationwide sixteen years cohort [12,13]. The purpose of this study is to perform a broad evaluation and description of the epidemiological profile, access to treatments and the main clinical outcome of the MM patients treated by SUS.

## Methods

### Study design and setting

This study employed a nationwide, non-concurrent, open cohort design, with patient follow-ups conducted from 2000 to 2015. Data were developed through deterministic-probabilistic linkage of the patient-centered registry within the Hospital Information System, Ambulatory Information System and Mortality Information System [14]. The Hospital Information System contains data on hospitalization from both public and private hospitals contracted by SUS. The High-Complexity Procedure Authorization subsystem of the Ambulatory Information System database contains all information about chemotherapy including records about the medical diagnoses for which treatment was prescribed using the International Classification of Diseases, Tenth Revision (ICD-10) codes.

The chemotherapy dispensations recorded in the database were decoded, listed, and cleaned to extract information regarding the protocols utilized. Treatment effectiveness was assessed by comparing outcomes of patients exposed to bortezomib-based regimens compared to those treated with other chemotherapeutic regimens.

Patients were categorized into therapeutic groups based on exposure to specific agents at any time during their treatment, regardless of treatment line. For instance, the ‘bortezomib-based’ group comprised all patients who received bortezomib at any point during the study period. This inclusive approach aimed to evaluate the overall impact of drug exposure across the disease trajectory.

**Figure 1:**
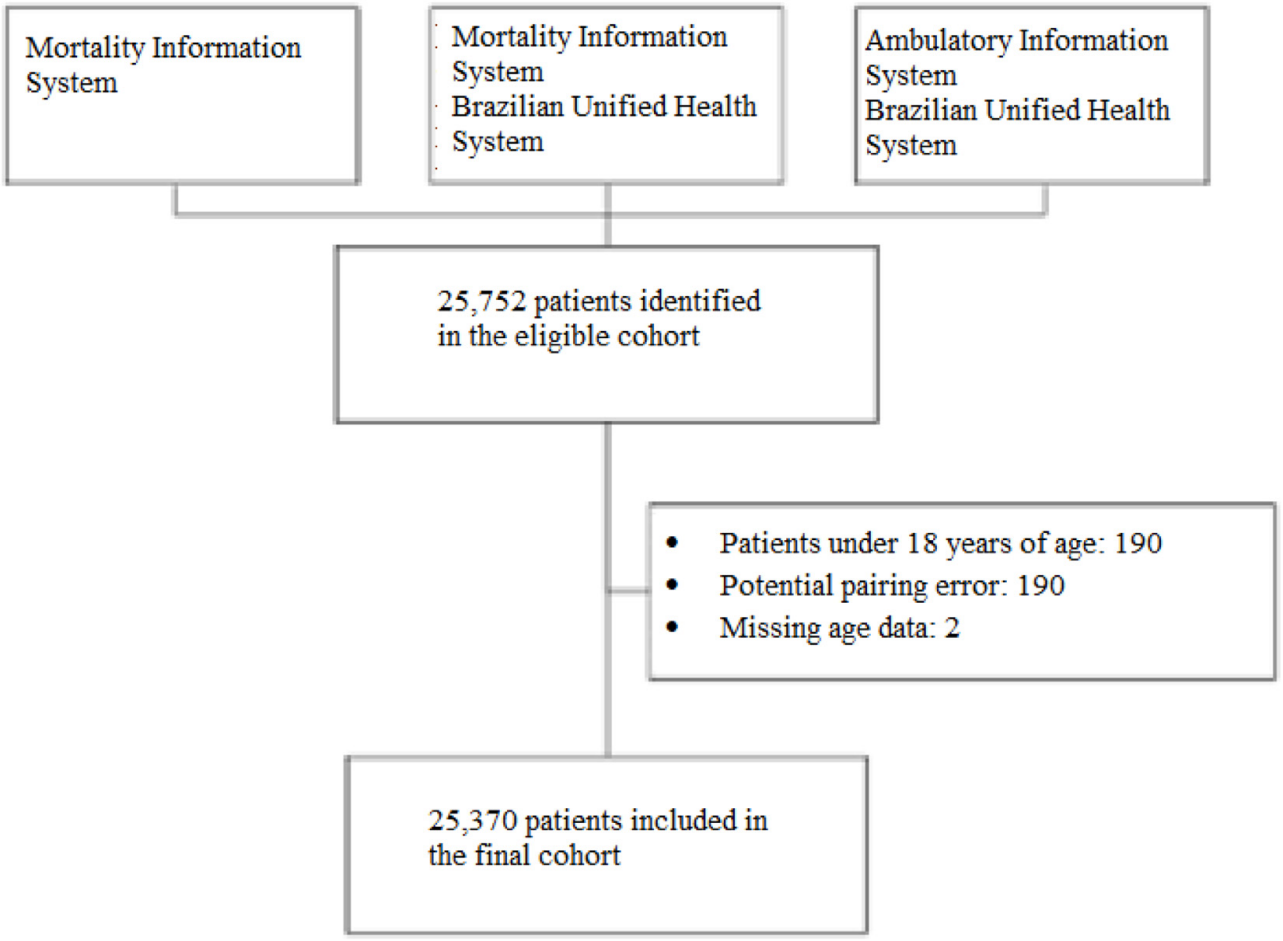
Cohort selection flow.

The study entry period was between January 2000 to December 2014, and patients were followed up from January 2000 to December 2015 (16 years). This strategy assured a minimum follow-up of 12 months. The inclusion criteria for this study were as follows: patients who received one or more treatments for MM (ICD C90.0), individuals over 18 years of age, and those initially treated between 01/01/2000 and 31/12/2014. Patients were censored if they abandoned or interrupted their treatment or at the end of follow-up (right censoring). Treatment failure events were characterized by death (Figure 1).

### Ethical aspects

The use of the National Database was evaluated and approved by the Research Ethics Committee of Federal University of Minas Gerais (CAAE - 16334413.9.0000.5149).

### Statistical analysis

The frequency distribution was analyzed for categorical variables. Measures of central tendency and variability were determined for numerical and quantitative variables (sociodemographic and clinical characteristics). Among other available variables, the chemotherapy regimen was used to stratify survival based on drug treatment.

The baseline was used as the first event (first chemotherapy or hospitalization for chemotherapy) to calculate the overall survival (OS). The Kaplan-Meier technique was used to determine the cumulative probability of survival of patients included in the study and according to the explanatory variables of the study. The Log-Rank test was used for subgroup analyses and test the hypothesis of equality between survival curves.

The proportional hazards model – Cox Model – was used to calculate hazard ratios (HR) and 95 % confidence intervals (95 % CI) of covariables that were statistically significant (*p*-value <0.05) in the Log-Rank test. The software “R” version 4.1.3, of R Foundation for Statistical Computing, Microsoft Excel® business 2019 was used for statistical analysis.

## Results

**Figure 2:**
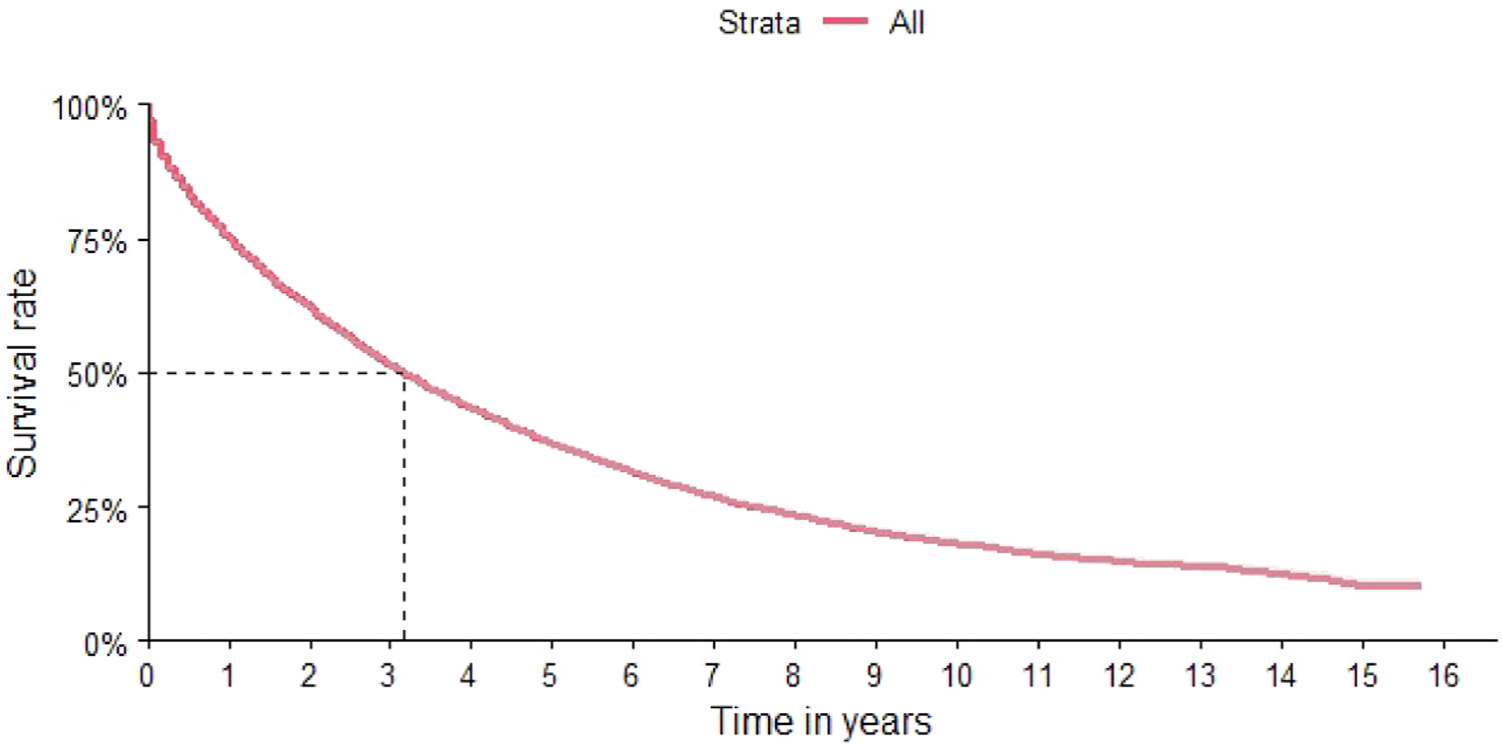
Overall survival.

The characteristics of the cohort are shown in Table 1. The final cohort consisted of 25,370 patients with 50.71 % being males. The median age was 62 years, with 70 % of patients over 56 years of age (Table 1). The distribution according to region identified a higher concentration of patients in the southeastern region (49.9 %), followed by the northeast and the south of the country (21.7 % and 19.3 %, respectively) (Table 1). The OS of the total study population was 37 months (95 % CI: 36–38 months) (Figure 2).

**Table 1:** Characteristics of the patients included in the cohort.

Variable	*n* = 25,370
**Sex – *n* (%)**	
Female	12,505 (49)
Male	12,865 (51)
**Age at baseline** - Median (IQR)	62 (54 to 71)
**Age range at baseline - *n* (%)**	
>65 years	10,122 (40)
18 - 25 years	103 (0.4)
26 - 35 years	408 (1.6)
36 - 45 years	1850 (7.3)
46 - 55 years	5089 (20)
56 - 65 years	7798 (31)
**Self-declared skin color - *n* (%)**	
Asian	258 (1)
White	8032 (32)
Indigenous	3 (<0.1)
Unknown	12,299 (48)
Brown	3879 (15)
Black	899 (3.5)
**Residence region at baseline - *n* (%)**	
Central-West	1703 (6.7)
North	635 (2.5)
Northeast	5596 (22)
South	4394 (17)
Southeast	13,042 (51)
**ICD10 Description at baseline - *n* (%)**	
Extramedullary plasmacytoma	604 (2.4)
Gammopathy	480 (1.9)
Multiple myeloma	23,833 (94)
Multiple myeloma and malignant plasma cell neoplasms	71 (0.3)
Plasma cell leukemia	382 (1.5)
**Cohort entry period - *n* (%)**	
2000 - 2003	6185 (24)
2004 - 2007	5306 (21)
2008 - 2011	7293 (29)
2012 - 2015	6586 (26)
**Medication at baseline - *n* (%)**	
bortezomib (Bortezomib) Based	445 (1.8)
Thalidomide Based	2633 (10)
Others	22,292 (88)
**Hematopoietic stem cell transplantation - *n* (%)**	
No	22,644 (89)
Yes	2726 (11)
**Comorbidity Charlson Index at baseline -** Median (IQR)	2.00 (2.00 to 3.00)
**Frailty Index at baseline** - Median (IQR)	0 (0 to 11)
**Mean time of illness before baseline** - Median (IQR)	0 (−1 to 0)
**Mean time in the cohort** - Median (IQR)	18 (6 to 40)
**Event type - *n* (%)**	
Censure	12,328 (49)
Death	13,042 (51)

**Figure 3:**
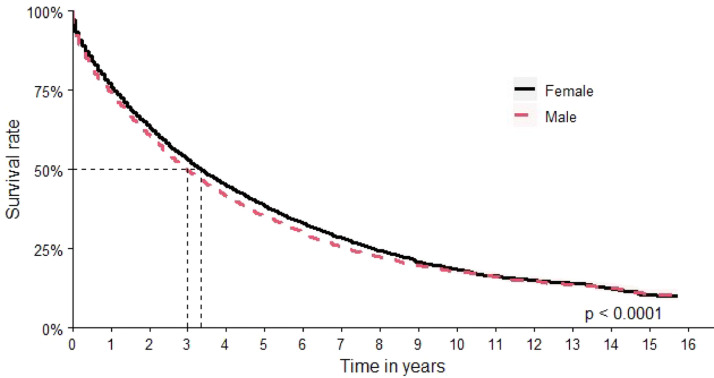
Overall survival by sex.

The assessment by sex found an OS of 40 months (95 % CI: 39–42 months) for women versus 36 months (95 % CI: 34–37 months) for men (Figure 3).

Table 2 presents the relative risk estimates (HRs) and corresponding 95 % CIs for the main sociodemographic and clinical characteristics analyzed in the cohort based on a multivariable Cox proportional hazards model. This table allows for the identification of groups with higher or lower risk of death within the cohort, contributing to the understanding of disparities in survival outcomes. In the univariate analysis, male sex was associated with an increased risk of death (HR: 1.12; 95 % CI: 1.08–1.16).

**Table 2:** Estimated risk rate according to the COX proportional analysis model for the total cohort (*n* = 25,370; deaths: 13,042).

Characteristic	HR	95 % CI	*p*-value
**Sex**			
Female	—	—	
Male	1.09	1.05–1.13	**<0.001**
**Age at baseline**	1.02	1.02–1.02	**<0.001**
**Age range at baseline**			
>65 years	—	—	
18–25 years	0.47	0.34–0.66	**<0.001**
26–35 years	0.50	0.43–0.59	**<0.001**
36–45 years	0.48	0.44–0.52	**<0.001**
46–55 years	0.66	0.63–0.70	**<0.001**
56–65 years	0.76	0.73–0.79	**<0.001**
**Self-declared skin color**			
Asian	—	—	
White	1.36	1.09–1.70	**0.007**
Indigenous	2.38	0.58–9.67	0.23
Unknown	2.26	1.81–2.83	**<0.001**
Brown	1.23	0.98–1.54	**0.080**
Black	1.15	0.90–1.47	0.25
**Residence region at baseline**			
Central-West	—	—	
North	0.94	0.82–1.08	0.38
Northeast	0.86	0.80–0.93	**<0.001**
South	1.12	1.04–1.21	**0.003**
Southeast	0.93	0.87–1.00	**0.052**
**ICD 10 Description at baseline**			
Extramedullary plasmacytoma	—	—	
Gammopathy	1.14	0.95–1.35	**0.15**
Multiple myeloma	1.25	1.12–1.41	**<0.001**
Multiple myeloma and malignant plasma cell neoplasms	1.26	0.92–1.72	**0.15**
Plasma cell leukemia	1.49	1.25–1.79	**<0.001**
**Cohort entry period**			
2000 - 2003	—	—	
2004 - 2007	1.22	1.16–1.28	**<0.001**
2008 - 2011	0.99	0.94–1.03	0.53
2012 - 2015	0.85	0.81–0.90	**<0.001**
**Medication at baseline**			
Bortezomib Based*	—	—	
Thalidomide Based	1.31	1.07–1.60	**0.009**
Others	1.71	1.41–2.06	**<0.001**
**HSCT**			
No	—	—	
Yes	0.36	0.34–0.39	**<0.001**
**Comorbidity Charlson at baseline**	1.06	1.05–1.07	**<0.001**
**Frailty Index at baseline**	1.00	1.00–1.00	**<0.001**
**Mean time of illness before baseline**	1.00	1.00–1.01	**<0.001**
**Mean time in the cohort**	0.95	0.95–0.95	**<0.001**

**Figure 4:**
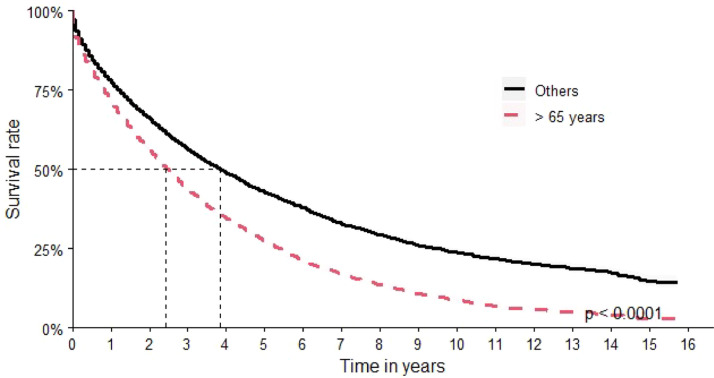
Overall survival by age.

Over 65-year-old patients had an OS of 29 months versus 44 months for the other age groups (Figure 4).

The risk of death for patients from the south of Brazil was the highest in the country (HR: 1.11; 95 % CI: 1.03–1.20) and the lowest risk of death was identified in patients from the northeastern region (HR: 0.84; 95 % CI: 0.78–0.91). The relative risks of the main characteristics evaluated in the study are shown in Table 2.

**Figure 5:**
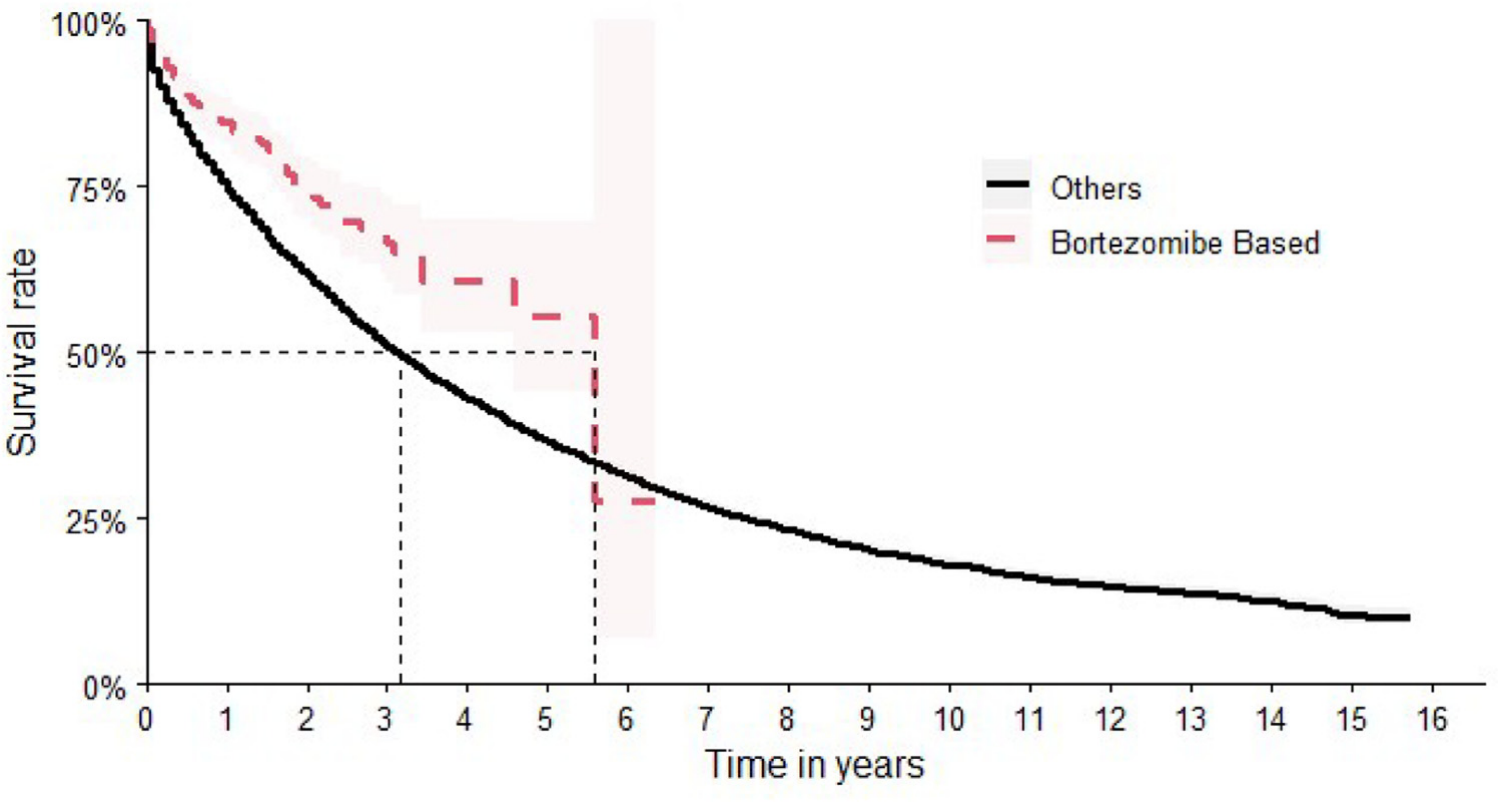
Overall survival comparing bortezomib-based chemotherapy with other regimens.

Although bortezomib had not been formally incorporated into the SUS at the time of the study, patients receiving therapeutic regimens containing bortezomib were nevertheless identified (*n* = 445 patients). In terms of OS, bortezomib-based chemotherapy showed the best results, achieving a median time of 67 months (95 % CI: 55-NA]). This corresponds to a Hazard Ratio of 0.60 (95 % CI: 0.50–0.73), indicating a significantly reduced hazard of death compared to other treatment regimens (Figure 5).

**Figure 6:**
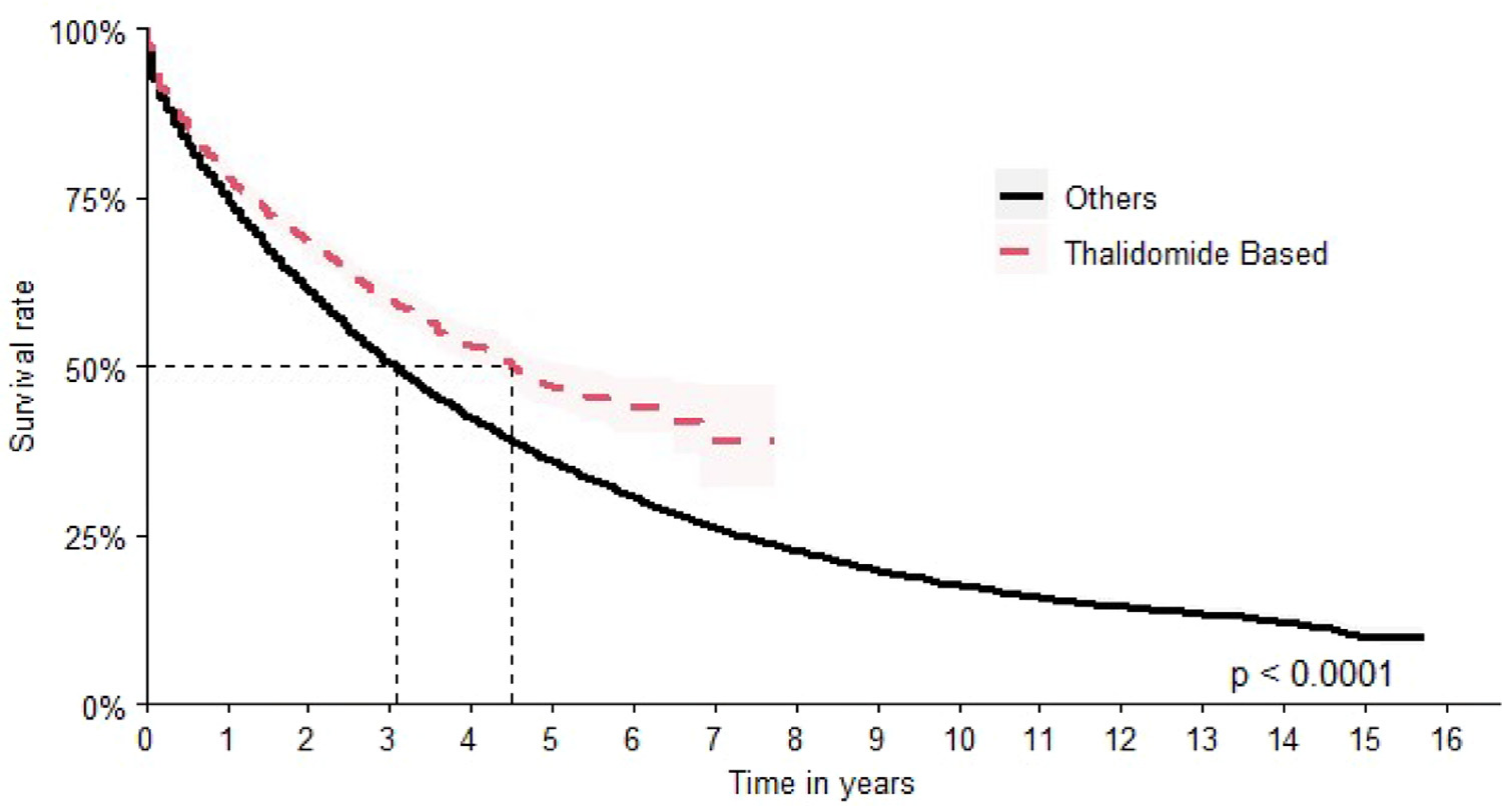
Overall survival for thalidomide-based chemotherapy.

The second most common scheme was thalidomide-based with a median OS of 54 months (95 % CI: 50–62) and HR 1.30 times better when compared to all other options (HR: 0.77; 95 % CI 0.72–0.82) (Figure 6).

**Figure 7:**
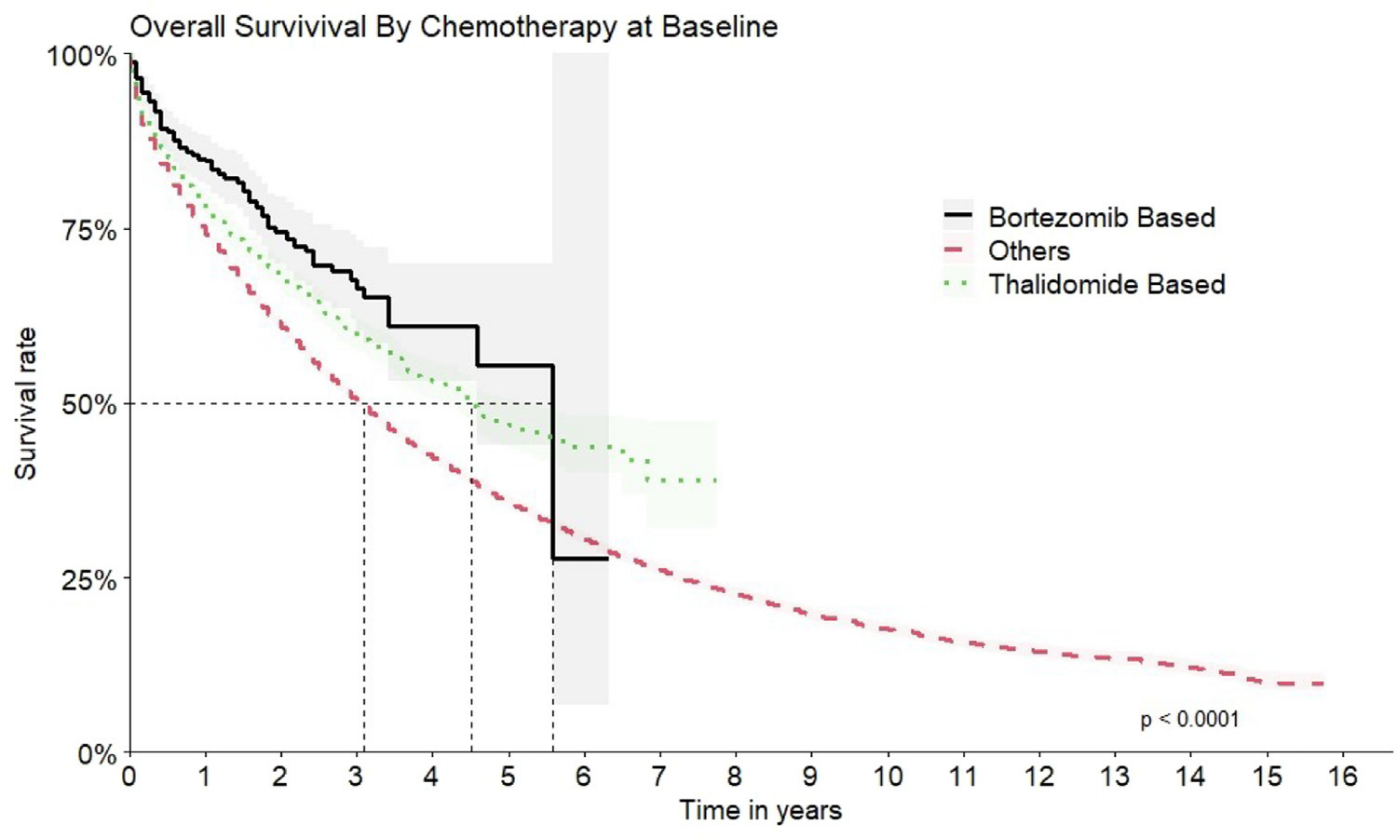
Overall survival by therapeutic regimen.

The comparison of the OS for all the therapeutic regimens is shown in Figure 7.

**Figure 8:**
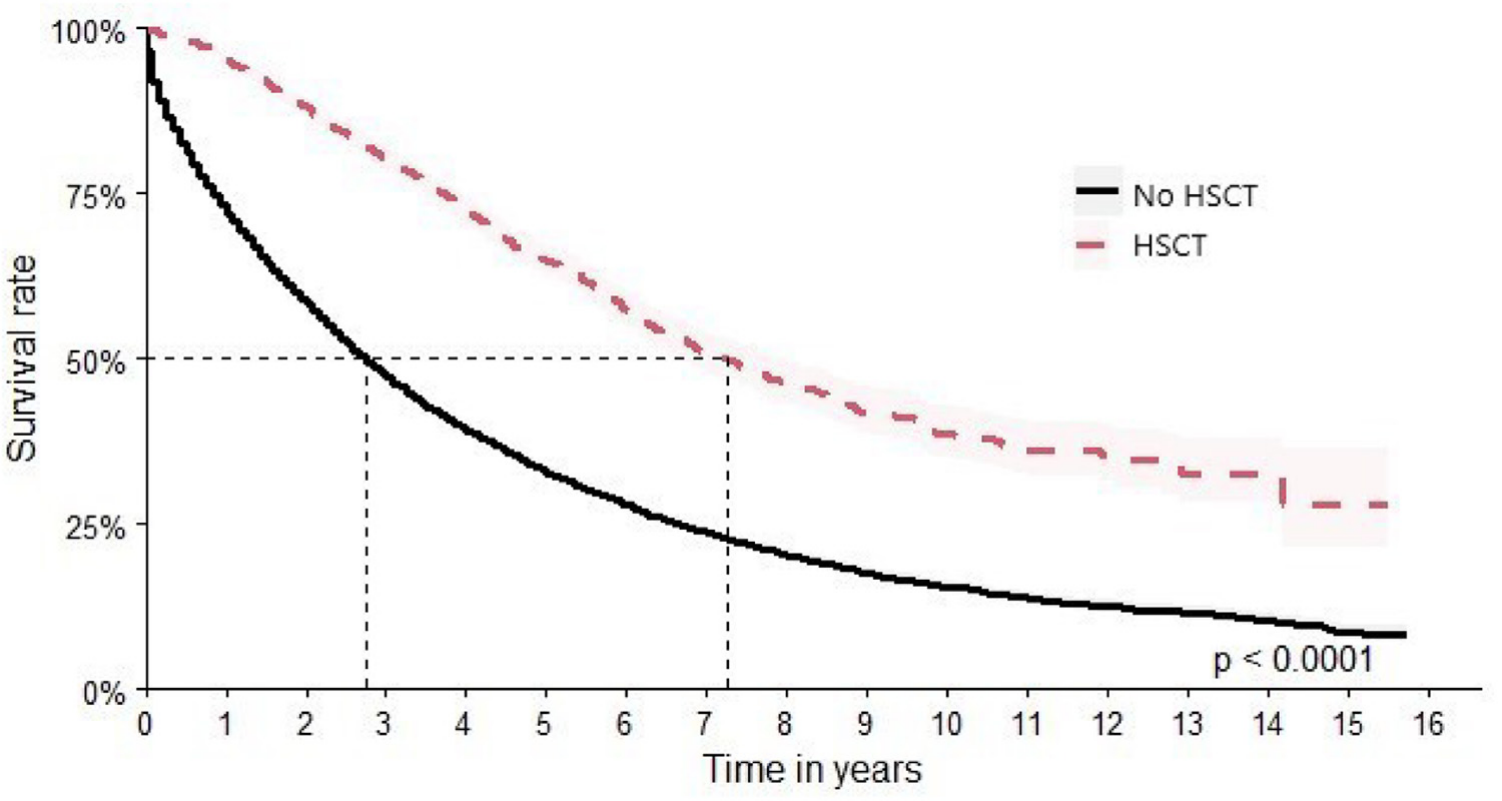
Overall survival after hematopoietic stem cell transplantation.

In this study, 2726 patients were identified as having undergone HSCT. This subgroup achieved a median survival time of 87 months (95 % CI: 81–95), and HR 1.51 times better (HR: 0.36; 95 % CI: 0.34–0.39) versus 33 months (95 % CI: 32–34) for patients who did not undergo HSCT (Figure 8).

## Discussions

MM is an onco-hematological neoplasm with a low incidence. The median age at start of treatment in this study was 62 years, which is consistent with the Brazilian literature. In a study conducted in Reginal Hospital in Mato Grosso do Sul of patients treated from January 2013 to December 2017, the median age of patients was 63 years, corroborating the findings of this study [15]. The same median (63 years; range: 37–82 years) was found in a study conducted by Silva et al. ^16^ in Clinical Hospital of Minas Gerais [16]. The variation found in this cohort was 18 to 98 years, with 70 % of patients being over 56 years old. The median age in the present study is comparable to the 60.5 years reported by Hungria et al. [5]. Given that SUS provides care for most of the population [17], with no significant access restrictions compared to the private healthcare system, our results are likely representative of the overall national profile of MM patients.

The age of patients at the beginning of treatment for MM is lower in Brazil than in other countries. In a study using data from the French health care system, the median age was 74 years (range: 63–81 years). In the United States, the median age at diagnosis was 69 years, with 60 % of patients over 65 [18]. In that report, there was no significant difference in incidence between sexes, but mortality was higher in men (HR: 1.12; 95 % CI: 1.08–1.16) [18]. In the United States, the incidence of MM was 1.5 times higher in men (2.1/100,000) than in women (1.4/100,000) and the mortality in 2018 was 59,000 deaths in men versus 47,000 in women, in the same period.

The life expectancy in Brazil in 2010 was assessed at 73.9 years, which can be considered lower than countries such as the USA, which had an approximate life expectancy of 80 years in 2010 [19,20]. Regarding the epidemiological profile of MM, the age at diagnosis found in this cohort is also lower when compared to patients in the USA (66–70 years), with 37 % of patients being younger than 65 years, as reported by Kazandjian [21].

The OS found in the current cohort reached a median time around three years (37 months), a result consistent with the literature, considering the same period [21]. Different factors affect the OS of MM patients in Brazil. Notably are the lack of access to or availability of newer medicines throughout the country and the low rates of autologous HSCT despite financing by SUS. The low rates of HSCT can be attributed to a combination of factors, including insufficient specialized medical centers, geographic disparities in healthcare access, long waiting times, and socioeconomic barriers that limit patient access to the treatment. The current study observed that transplant-eligible patients exhibited a longer OS when compared to their non-transplanted counterparts, aligning with findings from other published studies [22,23].

Eligibility to HSCT is the best prognostic factor in MM; data obtained in this study are compatible to data from the International Myeloma Working Group in five countries in Latin America, where OS of HSCT-eligible patients was 73.6 months versus 43.0 months for ineligible patients [24].

According to Moore et al. [25] the incidence of MM is on the rise in Nordic countries and other Western nations. Despite this demographic change, the inclusion of individuals from the older age group in clinical trials can be a challenge as evidenced in studies such as VISTA [26], FIRST [27], ALCYONE [28] and MAIA [29]. Over 65-year-old patients often present clinical conditions that hinder their participation in clinical trials, particularly due to frailty and the complexities involved in testing new therapies. In this context, real world evidence becomes relevant, as it reflects outcomes in the actual MM population, taking into account the age, sex, and other factors [25].

The improvement in OS following the incorporation of novel agents has been well described in the literature. In this study, an OS of 54 months was observed among patients who received thalidomide in the therapeutic regimen. Two studies evaluated the regimen of melphalan and prednisone with or without thalidomide in previously untreated patients and elderly patients. The study by Hulin et al. [30] in over 75-year-old patients with early MM, reported an OS of 45.3 months versus 27.7 months. The study conducted by Facon et al. [31] of over 65-year-old patients showed an OS of 51.6 months versus 33.2 months in the group without thalidomide. These studies reinforce the finding of the benefits of associating thalidomide to the therapeutic regimen and the difference in OS regarding age at diagnosis [30,31].

Thalidomide was market approved in Brazil for MM treatment in 2000 and was integrated into the SUS during the study period [32]. The low percentage of patients using this drug may be due to the need for patient monitoring and guidance, particularly in a vast country like Brazil, where access to Thalidomide and HSCT is more limited outside major urban centers.

In a study conducted by Hungria et al. in five Latin American countries, HSCT was performed in 58.6 % of the patients for whom it was initially planned, and in only 26.9 % of the total patient population [24]. Despite the observed benefits in treatments involving thalidomide or HSCT, and their availability in the SUS, physicians and medical institutions have the possibility to choose which treatments to prescribe for MM patients. The guideline that enumerates the available procedures is not obligatory, leading to variations in therapy access based on the clinical judgment of the medical team.

The median OS for patients using bortezomib was 67 months versus 37 months in the total study population. In the Phase 3 ENDEAVOR study of relapsed or refractory over 18-year-old patients using bortezomib and dexamethasone (Vd), the OS was 40 months [33]. In the VISTA study, previously untreated patients using an association of bortezomib, melphalan and prednisone (VMP), OS was 56.4 months over a five-year follow-up [34], supporting what has already been discussed regarding the increased OS related to the early use of technology.

A key methodological consideration is that patients were classified according to exposure to specific therapeutic agents at any point during the treatment course, rather than being limited to first-line therapy. This methodological choice aimed to assess the overall impact of drug exposure on patient survival across the entire disease trajectory. Although this approach does not allow for the isolation of the effects of bortezomib when used exclusively as first-line treatment, it better reflects the real-world complexity of therapeutic regimens and captures the cumulative benefit associated with access to effective agents. The improved OS observed among bortezomib-exposed patients may partially reflect treatment selection bias and the advantage of longer survival allowing access to subsequent lines of therapy. However, the findings suggest that bortezomib exposure, regardless of treatment line, is associated with favorable survival outcomes. Future studies designed to evaluate line-specific treatment effects are warranted to further elucidate the role of bortezomib in different therapeutic stages.

The Brazilian National Committee for Technology Incorporation (CONITEC) carefully carries out and deliberates on the continuous assessment of new technologies, costs, and equity in access to healthcare. This process considers several factors, such as effectiveness, safety, cost-effectiveness, and epidemiological needs. However, Bortezomib was only formally incorporated by the CONITEC into the SUS in 2020 thereby explaining the low number of patients treated with this drug in this cohort [9,10].

However, prior to this formal incorporation some factors such as approval for market entry by the national regulatory agency (National Health Surveillance Agency - ANVISA) with its scientific evidence of efficacy, encouraged its use by physicians. Another reason is the model of finance of the oncology service providers in Brazil where certain flexibility is allowed for when prescribing chemotherapy. SUS makes a fixed payment for patient treatment and oncology services providers are free to choose among therapeutic options between approved medicines. Despite the significant progress made by SUS in expanding access to a broad range of therapeutic options, there is still a need for more timely decisions by CONITEC [35] regarding the incorporation of new oncology drugs. Accelerating this process, while maintaining rigorous evaluation criteria, could improve access and reduce delays in the availability of innovative treatments. Litigation about oncology treatments is a major issue in Latin America, especially in Brazil and a faster assessment would reduce the conflict. In the case of Bortezomib, an earlier incorporation into SUS, coupled with adequate funding for oncology services, could have potentially saved lives, given the observed impact on OS in the current study compared to the treatments available at that time within SUS.

## CRediT authorship contribution statement

**Deborah Marta do Santos Oliveira:** Formal analysis, Investigation, Methodology, Software, Writing – original draft. **Isabela Cristina Menezes de Freitas:** Formal analysis, Methodology, Visualization, Writing – review & editing. **Wallace Mateus Prata:** Formal analysis, Methodology, Visualization, Writing – review & editing. **Isabella Zuppo Laper:** Formal analysis, Investigation, Methodology, Software. **Pâmela Santos Azevedo:** Formal analysis, Investigation, Methodology, Software. **Adriano de Paula Sabino:** Conceptualization. **Marisa Yurico Itonaga:** Conceptualization. **Carmino Antonio de Souza:** Conceptualization. **Mariângela Leal Cherchiglia:** Conceptualization. **Juliana Alvares Teodoro:** Conceptualization, Supervision. **Francisco de Assis Acurcio:** Conceptualization, Supervision. **Augusto Afonso Guerra Junior:** Formal analysis, Investigation, Methodology, Project administration, Software, Supervision, Visualization, Writing – review & editing.

## Conflicts of interest

The author declares no conflicts of interest.
